# Discovery of Unexpected Sphingolipids in Almonds and Pistachios with an Innovative Use of Triple Quadrupole Tandem Mass Spectrometry

**DOI:** 10.3390/foods9020110

**Published:** 2020-01-21

**Authors:** Federico Maria Rubino, Michele Dei Cas, Monica Bignotto, Riccardo Ghidoni, Marcello Iriti, Rita Paroni

**Affiliations:** 1 Dipartimento di Scienze della Salute, Universita’ degli Studi di Milano, I-20142 Milano, Italy; federico.rubino@unimi.it (F.M.R.); michele.deicas@unimi.it (M.D.C.); monica.bignotto@unimi.it (M.B.); riccardo.ghidoni@unimi.it (R.G.); 2 Aldo Ravelli Center for Neurotechnology and Experimental Brain Therapeutics, Dipartimento di Scienze della Salute, Universita’ degli Studi di Milano, I-20142 Milano, Italy; 3 Dipartimento di Scienze Agrarie e Ambientali—Produzione, Territorio, Agroenergia, Universita’ degli Studi di Milano, I-20133 Milano, Italy; marcello.iriti@unimi.it

**Keywords:** ceramides, lipids, functional food, mass spectrometry, nutraceuticals, traditional food

## Abstract

The densely packed storage of valuable nutrients (carbohydrates, lipids, proteins, micronutrients) in the endosperm of nuts and seeds makes the study of their complex composition a topic of great importance. Ceramides in the total lipid extract of some ground almonds and pistachios were searched with a systematic innovative discovery precursor ion scan in a triple quadrupole tandem mass spectrometry, where iso-energetic collision activated dissociation was performed. Five descriptors were used to search components with different C18 long chain bases containing different structural motifs (d18:0, d18:1, d18:2, t18:0, t18:1). The presence of hexoside unit was screened with a specific neutral loss experiment under iso-energetic collision activated dissociation conditions. The discovery scans highlighted the presence of two specific hexosyl-ceramides with a modified sphingosine component (d18:2) and C16:0 or C16:0 hydroxy-fatty acids. The hexosyl-ceramide with the non-hydroxylated fatty acid seemed specific of pistachios and was undetected in almonds. The fast and comprehensive mass spectrometric method used here can be useful to screen lipid extracts of several more seeds of nutraceutical interest, searching for unusual and/or specific sphingosides with chemically decorated long chain bases.

## 1. Introduction

The densely packed content of carbohydrates, lipids, proteins, and other micronutrients in the endosperm of nuts and seeds is the storage of nutrients for the development and early growth of the seed into a new plantlet [1,2]. This characteristic makes them a complete and healthy food for animals and humans. Therefore, due to their traditional importance as a food staple in many countries and as a highly prized nutraceutical complement of health-promoting diets and of value-added traditional foods, the study of their composition is a topic of great importance [3]. Composition studies are especially important to relate the contents of micronutrients to health status [4,5] and to confirm the presence of specific components in complex preparations [6].

In particular, the ceramides present in the fatty component of nuts display a degree of chemical diversity in the sphingoid bases, fatty acid substituents, and C-1 appendages [7]. Limited information is available on occurrence and levels of ceramides in nuts. Miraliakbari and Shahidi et al. [8] reported that almond and pistachio oils contain 240 and 330 mg/100 g of total sphingolipids, respectively, measured utilising TLC-FID as a non-specific method of quantification. Using 750 Da as a mean representative value for sphingolipid molecular masses, this value corresponds to approximately 500 nmol/100 g. By using LC-MS/MS, we found 200 pmol/g = 20 nmol/100 g of ceramide species with the “standard” (i.e., mammalian) sphingoid base, 1,3-dihydroxy-D4-C18 sphingosine [9]. Even accounting for inefficient extraction and unspecific measurement, this discrepancy suggests that there is a major pool of sphingolipids unaccounted for in nuts.

The measured “standard” ceramides are not the only derivatives of sphingoid bases that are currently known to occur in nuts. Sang et al. [10] used high-resolution 1D and 2D NMR data to identify in almonds a monoglucocerebroside with a modified sphingoid base, sphinga-4,8-dienine. Fang et al. [6] employed liquid chromatography coupled to electrospray mass spectrometry with in-source fragmentation to identify further compounds in several plants, among which were almonds. Among those identified in almonds, there are ceramides and cerebrosides with trihydroxy bases with zero or one double bond, mainly 4-hydroxy-8-sphingenine (t18:1), amide-linked to very long chain fatty acids, with or without an *α*-hydroxy group. In this study, cerebrosides, expressed as d18:2-C16:0H-GLU, were measured at 68 µg/g (approximately 100 nanomoles/g, or 10 µmoles/100 g); thus, at a level that is at least two orders of magnitude higher than sphingosine d18:1 ceramides [6].

To systematically search for unexpected ceramides in lipid extracts, we propose an innovative use of the triple quadrupole tandem mass spectrometry based on a precursor ion scan with iso-energetic collision activated dissociation, aimed at systematic discovery and preliminary characterization of the main sphingosine components of almond and pistachio. Specifically, we searched for components with different C18 long chain bases containing different structural motifs (d18:0, d18:1, d18:2, t18:0, t18:1). The presence of hexoside unit was screened with a specific neutral loss experiment under iso-energetic collision activated dissociation conditions. Connectivity confirmation was achieved by fragment ions analysis with accurate mass measurements.

## 2. Results

### 2.1. Systematic Discovery of Sphingolipids in Almonds and Pistachios

We modified our standard LC-MS-MS method used for targeted ceramide measurement [9] to allow the systematic identification (discovery) of ceramides and ceramide hexosides with different sphingosine and fatty acid components. Modifications include:

(a) The use of full scan Precursor Ion (PI scan) of five O” ceramide reporter fragments (*m*/*z* 262, 264, 266, 280, 282; Table 1) to highlight ceramides with modified sphingosines;
(b) The use of a full scan Neutral Loss of C_6_H_12_O_6_ hexose (180.2 Da) to ascertain whether the putatively highlighted ceramides have a hexose attached unit;
(c) The use of a collision energy ramp synchronized to the scan of Q1 in the PI and NL modes (iso-energetic Precursor Ion, *i*-PI, and iso-energetic Neutral Loss, *i*-NL) to analyze all ceramides at the same value of effective collision energy (see Appendix A);
(d) An extended isocratic step (in respect to the previously published conditions [9]) at full gradient strength during the UPLC analysis of sphingolipids (total analysis time 22 min) to investigate the possible presence of ceramides with much longer fatty acids.

The systematic search for ceramides and hexose-linked ceramides that contain each of the five hypothesized LCBs was performed, for each sample, in four separate chromatographic runs. Each run alternates two 1-s *i*-PI scans of *m*/*z* 264 (reporter ion of d18:1 internal standards and of ceramides with d18:1 sphingosine) and one for each of the other LCBs (*m*/*z* 262, 266, 280, 282). This procedure allowed for an estimation of the amount of unexpected ceramides and carbohydrate-linked ceramides with reference to those of the Cer12:0 and Cer12:0-Glc that show in the *i*-PI scan of *m*/*z* 264.

To verify whether any identified compound is really a ceramide hexoside (there is no possibility to ascertain hexose stereochemistry and configuration with this set of experiments), an iso-energetic Neutral Loss scan (*i*-NL) of the hexose fragment (C_6_H_12_O_6_, MW 180.2 Da) was paired to the Precursor Ion scan that yielded the putative ceramide hexoside. 

**Figure 1:**
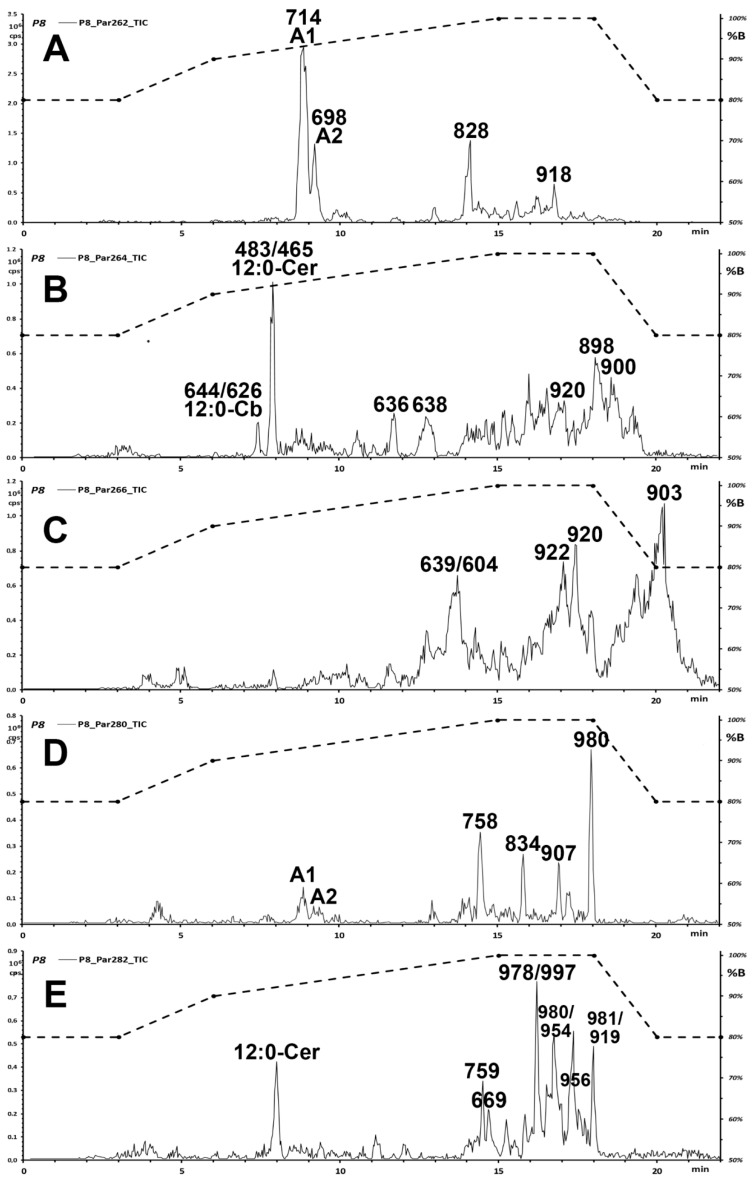
Chromatographic traces of the iso-energetic Precursor Ion scans of five long chain bases ((**A**–**E**), see Table 1 for their structures) in the extract of a pistachio cultivar. The dashed trace displays the chromatographic gradient, as volume fraction of acetonitrile (%B; right vertical axis). Numbers on top of prominent peaks are the *m*/*z* of the most intense ion signals. Labelled are the added internal standards (12:0-Cb and 12:0-Cer) and the newly identified hexosyl-ceramides A1 and A2.

Figure 1 shows the results of this set of experiments applied to a few interesting samples used to illustrate the discovery procedure. The other samples showed a similar pattern, with only differences in the relative intensity of the chromatographic peaks. 

**Figure 2:**
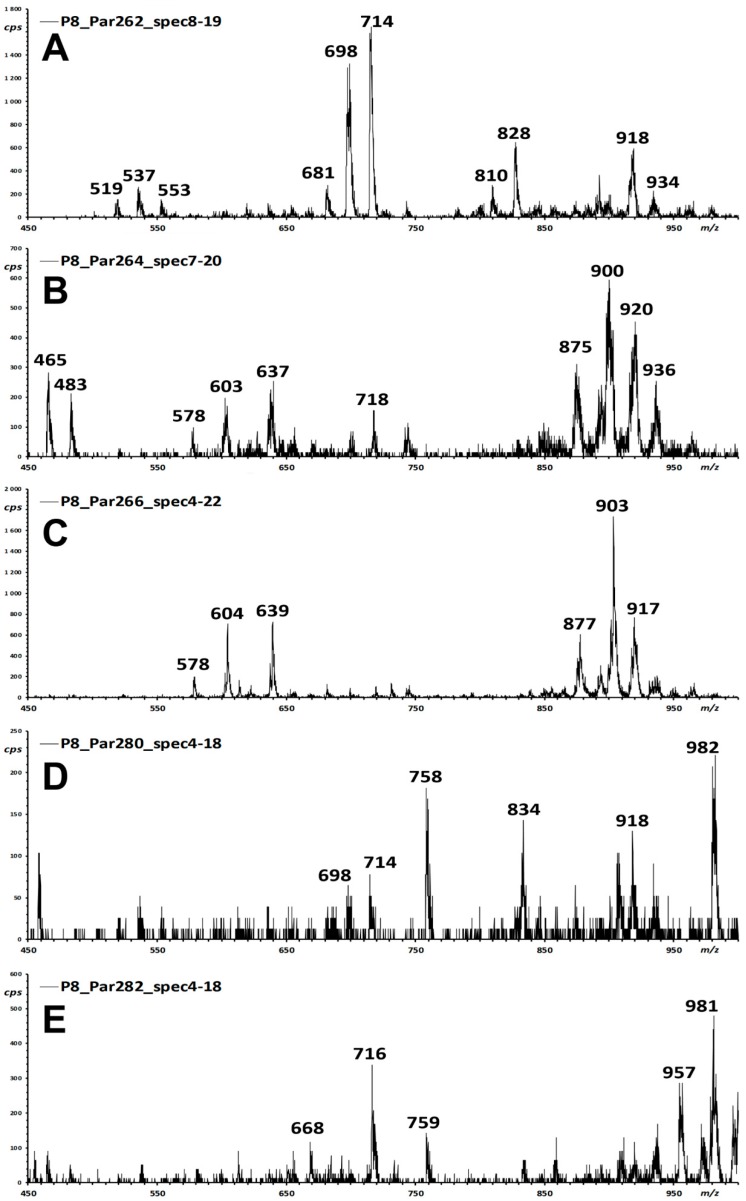
Integrated Precursor Ion mass spectra of the iso-energetic scan five long chain bases ((**A**–**E**), see Table 1 for their structures) in the extract of a pistachio cultivar. Integration is performed, for all experiments, within the 3.5 to 21 min interval of the chromatographic traces of Figure 1. Labelled are the *m*/*z* values of some main signals.

Figure 2 illustrates the corresponding integrated mass spectra of each iso-energetic Precursor Ion scan of Figure 1. Main signals that may correspond to likely unexpected ceramide species awaiting identification are highlighted.

The measurement of more than 50 chromatographic runs for the 15 examined samples highlighted the presence of at least two main chemical species. A major, earlier-eluting one (A1, retention time, RT ≈ 8.5 min; relative retention time, RRT = 1.18 vs. 12:0-Cb; MH^+^ 714) is present in all pistachio and almond samples (Figure 1A is a representative pistachio sample). A minor, later-eluting one (A2, RT ≈ 9.1 min; RRT = 1.23 vs. 12:0-Cb; MH^+^ 698) is present only in pistachios, but not in almonds. The two compounds featured prominently in the Par262 trace (Figure 1A) and yielded weaker peaks in the Par280 (Figure 1D) trace.

**Figure 3:**
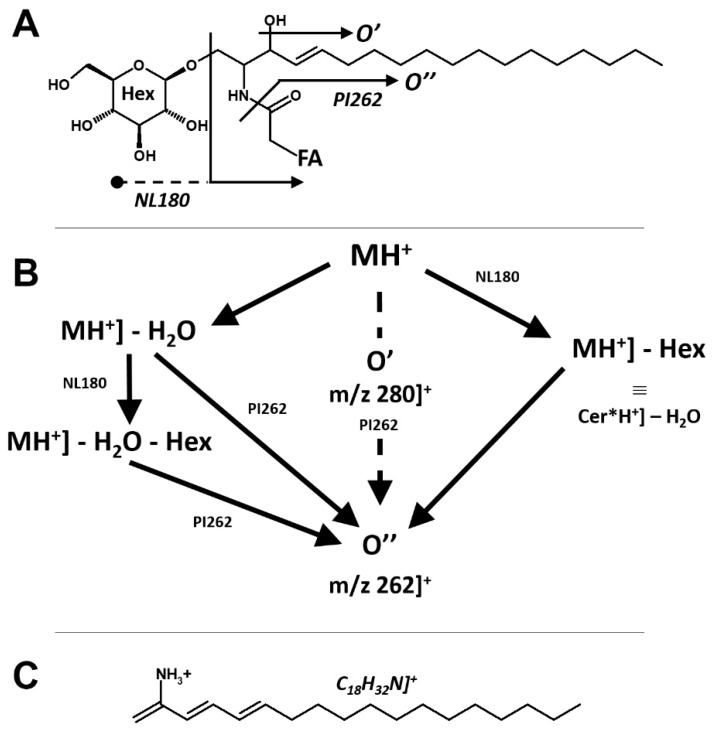
Overall fragmentation pattern of protonated hexosyl-ceramides, exemplified for those with the “standard” 1,3-dihydroxy-C-18:1 long chain base. Panel (**A**): correspondence of fragments to motifs in the molecular structure. Panel (**B**): development of decomposition processes and triple quadrupole scan experiments used to highlight individual molecules from their fragmentation. Panel (**C**): formal connectivity of the ceramide pivot fragment O’’.

**Figure 4:**
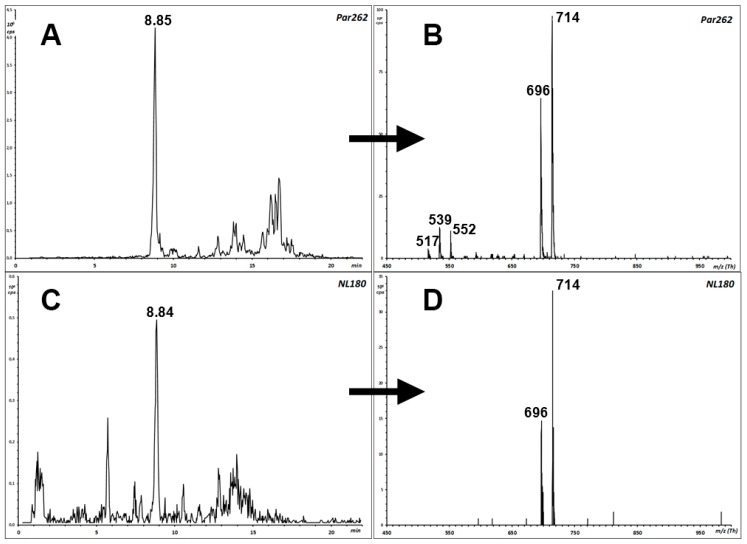
Confirmation of an unexpected hexose-containing ceramide species in the extract of an almond cultivar. The highlighted peak in panels (**A**,**C**) is that of compound A1. *m*/*z* 714 is the molecular mass (MH^+^) in both experiments (Par262, panel (**B**), and NL180, panel (**D**). *m*/*z* 696 derives from MH^+^ by water loss (panels (**B**,**D**)). In Par262 (panel (**B**)) *m*/*z* 552 and 534 derive from the previous ones by the loss of hexose.

According to the general fragmentation pattern of protonated ceramides reported in Figure 3 [11], the glycosidic nature of compounds A1 and A2 is warranted by the coelution also in the NL180 experiment (Figure 4A,C) and by the observation of the same precursor ions in the NL180 and Par262 spectra (Figure 4B,D).

**Figure 5:**
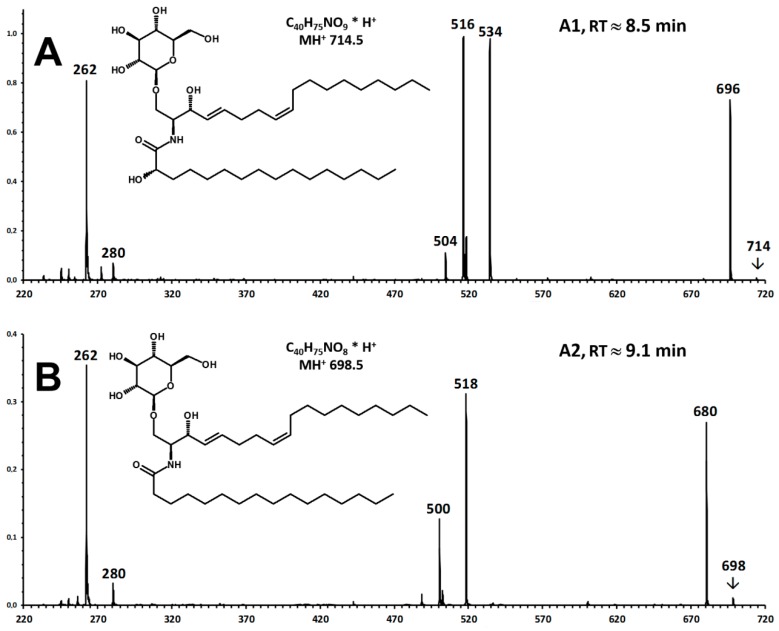
Enhanced Product Ion (EPI, CE 30 DV) spectra of unexpected ceramide species in the extract of a pistachio cultivar. (**A**) Compound A1 is that eluting at 8.5 min in the chromatographic trace A of Figure 1; (**B**) compound A2 is that eluting at 9.1 min.

Their connectivity was investigated by recording their Fragment Ion spectra in the EPI mode (Q3 used as a LIT) in separate chromatographic runs (A1, A2, Figure 5).

According to the fragmentation pattern summarized in Figure 3, compounds A1 and A2 thus consist of:

- A modified sphingosine that carries a further unsaturation in the C18 chain (d18:2, fragment ion at *m*/*z* 262 Th);
- The C-1 of sphingosine is linked to a hexose (paired losses of 180 Da from MH^+^ and [MH − H_2_O]^+^ ions);
- The 2-amino group of the sphingosine is linked to two saturated C16 fatty acids (mass difference between *m*/*z* 262 and the fragments generated by hexose loss), one of which (earlier-eluting compound A1) carries an additional hydroxyl group.

The occurrence of a linked hexose unit at C-1 was confirmed by performing an NL-180 scan concurrent to the Par262 scan, observing peaks in both traces at the retention times of the two compounds and MH^+^ and [MH − H_2_O]^+^ ions (Figure 3).

Final evidence of the identity of the precursor and fragment ions detected in the triple quadrupole instrument was achieved by re-analyzing two almond and two pistachio extracts under comparable chromatographic conditions in a quadrupole-ToF instrument that measures *m*/*z* at a resolution close to 30,000. Figure S1 and Table S1 summarize the results. High-resolution measurements match the elemental composition of the examined precursor ions and of the expected fragments within 2–26 ppm, thus confirming the interpretation of the LIT fragment ion spectra of Figure 4 and the connectivity of the two compounds.

Previous researchers already extracted and identified similar compounds in almonds [12]. The authors assigned the connectivity of the additional long chain base double bond as z-∆8 and identified the stereochemistry of the appended hexose with the extensive use of Nuclear Magnetic Resonance experiments.

### 2.2. Levels of the Discovered Sphingolipids in Almonds and Pistachios

The analysis of three almond and nine pistachio samples highlighted that A2 (which contains a saturated C16 fatty acid) occurs in both almonds and pistachios, while A1 (which contains a hydroxylated C16:0-h fatty acid) is specific of pistachio. Their abundance in the sample could be estimated in the absence of authentic purified reference ceramides, by comparing the peaks areas to that of C12:0 cerebroside (peak at 7.3 min in panel B of Figure 1) added as an internal standard. In pistachios, components A1 and A2 were estimated on the average at 19.3 and 5.2 µg/g of extracts, respectively. In almonds, compound A1 is estimated on the average at 24.2 µg/g of extracts. The levels of the two characteristic ceramides in the individual nut samples are reported in Table 2. 

While Reisberg and collaborators [12] did not perform an analytical assessment, but rather isolated and characterized component A1, they could purify approximately 120 mg from 14 kg of almonds. This amount corresponds to 8.6 µg/g and is close to that measured in our samples.

### 2.3. Interference of Triglycerides in the Discovery of Ceramides in Almonds and Pistachios

As apparent from the chromatographic traces and integrated mass spectra of Figure 1 and Figure 2, an intense signal was detected for materials that eluted later than 15 min and that contained arrays of signals up to the *m*/*z* 1000 limit of the Q1 quadrupole scan. The time limit of 15 min is the elution time of the ceramide with the longest (C24:0) fatty acid of the standard series. Grounding on the specificity of the even-*m*/*z* O” reporter fragments for nitrogen-containing molecules, this region of the chromatograms was explored to detect possible sphingoid compounds with more complex structures. 

In particular, the Par264 trace (Figure 2B) featured even-*m*/*z* molecular signals with a molecular mass around 900 Da (*m*/*z* 898, 900, and 902). Possible connectivity at this molecular size that is compatible with a ceramide structure might belong to epidermosides, a class of very large ceramides with a ω-hydroxylated fatty acid, to which a further fatty acid is connected through an ester bond. Such compounds are found in skin, as components of the lipid mixture of the stratum corneum [13]. However, the Fragment Ion spectra recorded for the main *m*/*z* signals did not match the expected behaviour for the anticipated structures. In particular, the observed fragmentation did not yield the fragment pair spaced by 18 Da that derives from the two fragmentation modes of the long chain ester bond. In addition, the compounds eluted earlier than expected for their molecular size based on the behaviour of the standard series of saturated and unsaturated sphingosine ceramides [14].

Since the occurrence of these likely spurious signals may bias the discovery of high-mass, unexpected sphingosine derivatives, a brief investigation was undertaken to understand their origin. In particular, the mass spectral features of the cluster at 18–20 min elution time in the Par264 experiment (Figure 1B and Figure 2B) matched the loss of the elements of fatty acid-ammonium ion pairs in the Product ion experiment (diglyceride fragments at *m*/*z* 599.5, 601.6, 603.5, data not shown), which is the well-documented behaviour of triglycerides [15]. High-resolution measurement in the Q-ToF mass spectrometer on the same extract confirmed the postulated identity of the observed signals as ammonium ion adducts of triglycerides (Figure S2). In particular, TG ammonium adducts at *m*/*z* 900.8 (TG 54:4) were mainly composed of dioleoyl-linoleoyl-glycerol (OOL). This identification is confirmed by the occurrence of the expected losses of fatty acids in the tandem mass spectra, with the generation of diglyceride fragments at *m*/*z* 601 and 603 in a 3:1 ratio (Figure S2). Under the adopted collision conditions, the acylium fragments do not form. 

To investigate whether co-extracted triglycerides may be the source of the high-mass signals erroneously identified as putative complex ceramide (Figure 1B and Figure 2B), a source scan at baseline unit resolution in Enhanced Mass Scan (EMS) mode was recorded (data not shown). Most *m*/*z* signals recorded at the retention times of the considered chromatographic peaks correspond to a cluster of eluting triglycerides (TG) with ^12^C isotopomers at *m*/*z* 896.6, 898.7, and 900.5. The observed ion clusters corresponded to ammoniated triglycerides with a total of 54 fatty acid carbon atoms and a number of unsaturations that range from six to four, or three, two, and one units of linoleic acid (*m*/*z* 896.6 TG 54:6, *m*/*z* 898.7 TG 54:5, *m*/*z* 900.5 TG 54:4). The precursor ions detected in the Par264 scan (Figure 1B and Figure 2B) at *m*/*z* 898.5, 900.5, and 902.6 correspond to the [M + 2] isotopomers of each ammoniated triglyceride (TG 54:6, TG 54:5, TG 54:4; Figure S2C,E), which mainly contain two ^13^C carbon atoms in the molecule. Therefore, the observed signal (Figure 1B and Figure 2B), that interferes with the O” reporter fragment ion of sphingosine ceramides, may correspond to the ^13^C_1_-isotopomer (*m*/*z* 264) of the acylium fragment of linoleic acid *all*-^12^C C_18_H_31_O^+^ (*m*/*z* 263) that is present as a minor process in the spectra of triglycerides ionized as ammonium-adducted species [16].

To test whether this hypothesis holds, the same sample was analyzed with a simultaneous recording of the Precursor Ion spectra of *m*/*z* 263 (the acylium fragment of linoleic acid, *all*-^12^C C_18_H_31_O^+^) and of *m*/*z* 264 (^13^C_1_-isotopomer). As apparent in the two superimposed graphs of Figure S3, the chromatographic profiles of *m*/*z* 263 and 264 closely match in the time frame later than approximately 9 min, and their intensities are in an approximately 4:1 ratio. Only in the time frame between 7.5 and 8.5 min, where the abundant 12:0 ceramides elute, the *m*/*z* 264 signal (the O” fragment of S18d:1) predominates over the background signal and yields the expected molecular precursors of the S18d:1 species. In the time frame later than approximately 9 min, the two Precursor Ion spectra record signals of ammoniated triglyceride and diglyceride from the bulk of the un-fractionated lipid extract (spectra not shown). In particular, between 18 and 20 min, the Par263 scan (Figure S3) detects domes of molecular signals, among which those at *m*/*z* 896.9, 898.8, and 900.9 (TG 54:6, TG 54:5 and TG 54:4). The corresponding Par264 scan detects domes of molecular signals (*m*/*z* 897–902, spectra not shown) with an intensity that is approximately 25% of that of the corresponding signals of the Par263 scan. This intensity ratio closely corresponds to that expected for the occurrence of the ^13^C isotopomers of the fatty acid in the fragment signals of the precursor triglycerides (Figure S4).

## 3. Discussion

Research on the characterization of oils and solid fats (butter) derived from nuts is extensive [17], due to the long traditional use of these products and of increasing consumption as alternatives to dairy sources. Most analyses focus on the composition of major components, such as the presence of fatty acids [18,19]), of triglycerides and of some minor components with nutraceutical properties, such as vitamins and antioxidants [8,20,21]. 

Sphingolipid research in plants has highlighted the presence of modified long chain bases, mainly t18:1 and d18:2, linked to commonly occurring and more rare odd-carbon and hydroxylated fatty acids [6,10,12,22,23]. 

Observation of ceramides with a modified long chain base and with specific fatty acids may increase the variety of chemical indicators that can be employed to confirm the presence of specific high-value nuts, such as pistachios and almonds, in food preparations, and to highlight contamination or adulteration with extraneous materials [2,24,25]. Several techniques are currently used for this task, including direct Near-Infra-Red [26] and Raman [27] spectroscopy, mineral element pattern [28], and chemometric evaluation of untargeted LC-HRMS analysis [29].

It is of note that selective scans in the Precursor Ion and Neutral Loss modes are unique of the tandem triple quadrupole (and, earlier, of some magnetic) mass spectrometer configuration. The sole limitation of the use of triple quadrupole is the unit resolution of the mass filters. As highlighted in our case, spurious signals can be generated from the unexpected contribution of isotopic components of bulk constituents. This approach allowed observing and understanding for the first time the interference of a high load of triglycerides in the selective discovery search for trace level nitrogen-containing lipophilic secondary metabolites by triple quadrupole tandem mass spectrometry methods. In addition, the comparatively lower sensitivity of the iso-energetic Precursor Ion approach is inherent to the use of a scan mode, rather than MRM, and is an unavoidable trade-off of the demonstrated higher coverage of different chemical species [30,31].

Complete characterization of the discovered ceramide hexosides entails recognition of the hexose stereochemistry and confirmation of the position and stereochemistry of the structural modifications of the fatty acid and of the sphingosine. These structural details cannot be easily differentiated by simple, *on the fly* recording of tandem mass spectra but need isolation of more substantial amounts of purified materials by mass spectrometry guided preparative methods. 

## 4. Materials and Methods

### 4.1. Reagents, Chemicals, and Standards

Sphingolipid standards, including ceramides with d18:1D4 sphingosine long-chain base (LCB) and saturated/singly unsaturated straight-chain even-carbon fatty acids (12–24 FA) and C12:0 cerebroside were purchased from Avanti Polar Lipids (Alabaster, AL, USA). Methanol, ethanol, acetonitrile, ammonium formate, and formic acid (all analytical grade) were supplied from Merck (Darmstadt, Germany). Water was MilliQ-grade (Millipore, Milford, MA, USA).

### 4.2. Plant Material

Three selected almond (A6, A7, A8) and nine pistachio (P7–P15) extracts were analyzed in this study. Their codes and their main characteristics are reported in Table 3.

### 4.3. Sphingolipid Extraction Procedure

The total lipid extracts were prepared as described elsewhere [9]. Briefly, after the addition of 10 μL of IS (Cer C12, gluCer C12 20 mM), the powder (250 mg) was extracted in a microtube with an O-ring seal screw with 500 μL of methanol, 100 μL water, and 250 μL chloroform [32]. The samples were sonicated for 30 min and incubated overnight in an oscillator bath at 48 °C. The supernatant was evaporated under a stream of nitrogen. The residues were dissolved in 150 μL of methanol and then centrifuged for 10 min at 13,000 rpm. The clean supernatant (130 μL) was transferred into the autosampler vials, and 10 μL were directly injected in LC-MS/MS.

### 4.4. LC-MS/MS Instrumentation

Two separate sets of mass spectrometry measurements were performed in different computerized integrated systems, each consisting of a liquid chromatograph coupled to a tandem mass spectrometer.

Discovery of ceramides and ceramide hexosides with a diverse long chain base was accomplished in a hybrid triple quadrupole-linear ion trap tandem mass spectrometer. The integrated liquid chromatograph system was an UltiMate^®^ 3000 LC Systems (Dionex™, Sunnyvale, CA, USA), with an autosampler, binary pump, and column oven (Thermo Fisher Scientific, Waltham, MA, USA). The tandem mass spectrometer was an AB Sciex 3200 QTRAP LC-MS/MS instrument with electrospray ionization (ESI) TurboIonSpray™ source (AB Sciex Framingham, MA, USA). Instruments were managed with manufacturers’ software (Analyst software (version 1.6.2) and according to manufacturers’ instructions. Results exported as .txt files were post-processed in custom spreadsheets, as needed. The principle of the innovative scan modes employed in this instrument is briefly described in Appendix A.

A set of confirmative measurements used a Shimadzu UPLC interfaced to a TripleTOF 6600 (Sciex, MA, USA) high-resolution hybrid quadrupole time-of-flight mass spectrometer equipped with Turbo Spray IonDrive, under essentially the same chromatographic conditions (*2.3*, *v. infra*). The resolution was close to 30,000 according to the manufacturer’s software evaluation. The main instrument parameters were: CUR 35, GS1 55, GS2 35, capillary voltage 5.5 kV, source temperature 350 °C, declustering potential (DP) 50 eV. Two analysis modes were applied. One (“*targeted*” approach) recorded, within each 1-s measurement cycle, the source spectrum (*m*/*z* 200–1400; 250 ms accumulation time) and the fragment ion spectra of selected precursors only (100 ms accumulation time). The other (“*untargeted*” approach) recorded, within each 1-s measurement cycle, the source spectrum, and the fragment ion spectra of the ten most abundant precursor ions that occur in the previous source spectrum. Collision energy was set at 30 V on a nitrogen gas target. Data were extracted with the proprietary PeakView data management software and exported to spreadsheets for further evaluation.

### 4.5. Separation and Detection of Sphingolipids by LC-MS/MS

Separation of the lipid extract containing ceramides was accomplished in an ACQUITY UPLC BEH C-8 Column, 130 Å, 1.7 μm, 2.1 mm × 100 mm (Waters, Millford, MA, USA) preceded by a security guard cartridge. The flow rate was 0.3 mL/min; the autosampler and the column oven were kept at 15 °C and 30 °C, respectively, the operating pressure was 450 Psi. 

The two mobile phases were: phase A, 2 mM ammonium formate in water and phase B 1 mM ammonium formate in methanol, both containing 0.2% formic acid (*v*/*v*). A multi-linear extended gradient with a total analysis time of 22 min was programmed: the column was equilibrated with 80% (B), increased to 90% (B) in 3 min, held for 3 min, increased to 99% (B) in 9 min, held for 3 min, back to the initial conditions in 2 min, and kept for 2 min at 80% (B). 

Mass spectrometry was performed in the positive ion mode (ESI+). The ion spray voltage was set at 5.5 kV, and the source temperature was set at 300 °C. Nitrogen was used as a nebulizing gas (GS 1, 45 psi), turbo spray gas (GS 2, 50 psi), and curtain gas (25 psi). Source spectra were recorded in separate experiments in the Enhanced MS (EMS) mode at a scan speed of 1000 Da/s that yielded baseline separation of unit mass peaks in the *m*/*z* range 450–1000. The collision-activated dissociation (CAD) MS-MS experiment used nitrogen as collision gas at the low pressure setting (1.2 × 10^−5^ Torr).

### 4.6. Untargeted Discovery LC-MS/MS Analysis by Iso-Energetic Precursor Ion and Neutral Loss Scan in a Triple Quadrupole

An untargeted discovery method was developed to investigate the presence of unexpected sphingolipids in the samples. Briefly, the method employs Precursor Ion (PI) scans of the analysing quadrupole (Q1), while holding the selecting quadrupole (Q3) to transmit the reporter fragment ions generated by CID of protonated ceramides with different long chain bases (fragment O”).

Those selected are at *m*/*z* 264.4 (d18:1D4 sphingosine, [11]) and isomeric d18:1D8 [6], *m*/*z* 262.4 (d18:2D4,8); *m*/*z* 280.4 (t18:1D8, [6]).

Another set of experiments employed Neutral Loss scans, whereby both Q1 and Q3 mass filters are simultaneously scanned at the same rate, with a fixed offset of transmitted *m*/*z*, which corresponded to the mass of a specific molecular unit. In this case, the neutral fragment was the C_6_H_12_O_6_ unit (MW 180.2) of hexose.

The upper scan range of Q1 was initially *m*/*z* 450–700 in 1 s (measurement of the standard ceramide panel), and later, the upper *m*/*z* value was raised to *m*/*z* 1000 to allow analysis of ceramides with as many 60 carbon atoms. During each 1-s scan of Q1 (or of linked Q1 and Q3), the CAD potential (q2-Q1) was synchronously ramped in order that, at each *m*/*z* value of the precursor ion transmitted by Q1, the effective collision energy was held constant (iso-energetic, or *i*-CID). The particular selected value of the effective collision energy was selected following a spectroscopic study of model ceramides. Actual employed values are of 1.6 eV for the Precursor Ion scans (generation of O” fragment from MH^+^) and 1.0 eV for the Neutral Loss scan (loss of the hexose unit from the MH^+^). Conversion of centre-of-mass collision energy to instrument voltage was accomplished with a re-arranged form of the standard equation (a more detailed explanation is reported in Appendix A). Typical ranges of collision voltage for the Precursor Ion scans were 27.3–44.5 DV for the low mass range (*m*/*z* 450–750) and 27.3–55.9 DV for the extended mass range (*m*/*z* 450–1000). The corresponding values for the Neutral Loss scan were 27.3–44.5 DV for the low mass range (*m*/*z* 450–750) and 27.3–55.9 DV for the extended mass range (*m*/*z* 450–1000).

### 4.7. Relationship of Molecular Structure to Chromatographic Retention

To assist and to confirm the identification of unexpected ceramide species, a descriptor of the relationship of molecular structure to chromatographic retention was established. A standard mixture of ceramides ranging from C 12:0 to 24:0 (2.6 µM each) was injected under described conditions. Natural logarithm of capacity factor *k’*, defined as the number of column void volumes necessary to elute each compound, was plotted against the natural logarithm of both the total number of carbon atoms in the ceramide and of the sole number of carbons of the FA (graph reported in Figure S5).

The void volume was estimated from the geometric parameters of the column and from the mobile phase flow. A correction factor for fractional column packing was identified from technical literature, as 0.51 of the physical column volume. Relative retention times (RRT) were calculated with reference to the elution of C12 glucosyl ceramide (cerebroside, 12:0-Cb for short).

### 4.8. Isotope Pattern Calculation

Isotope pattern calculation was accomplished with an online freeware calculator (https://www.envipat.eawag.ch/index.php; last accession 4 November 2019). The software allows modulation of the mass resolution to envision the profile of the isotope cluster in the employed instrumental conditions. In this instance, the resolution was modulated at two different values, according to the employed mass spectrometer. A value of 500 (DM/M), slightly lower than that effective in the triple quadrupole instrument, but sufficient to match the measured ion profiles, was employed to simulate the isotopic envelopes for the first tier of measurements. A value of 10,000 (DM/M) was employed to calculate the abundances and accurate *m*/*z* of precursor and fragment ions anticipated for the identified compounds, and to compare with high-resolution measurements.

## 5. Conclusions

This report furthers our previous one on the targeted analysis of ceramides (d18:0, d18:1) in almond and pistachio [9]. This technique exploits the performance of the triple quadrupole tandem mass spectrometer well beyond its traditional use by analytical chemists.

Success with this approach will prompt a complete investigation of the structural details of the observed phytochemicals that cannot be resolved with the analytical and spectroscopic techniques employed in this preliminary survey. Semi-preparative chromatographic separation of crude lipid preparations driven by the selective scan will enable to obtain enriched fractions of the unexpected components. The use of high-energy collision induced dissociation will characterize the connectivity of the ceramide and fatty acid subunits [11], and deglycosidation will enable to separately identify the linked hexose.

Availability of a fast and comprehensive method to screen lipid extracts for trace sphingosides with chemically decorated long chain bases will be applied to analyze several more seeds of a nutraceutical interest that are the base of typical foods of the Italian lore.

## Figures and Tables

**Table 1:** Structures of the screened long chain bases and of the corresponding reporter ion fragments.

ID	Core Structure of Long Chain Base (LCB)	LCB (MW) and O” (*m*/*z*)
**A**	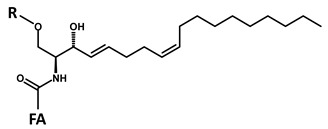	**d18:2-4,8** **C_18_H_35_NO_2_** **MW 297.49** **O”** **C_18_H_32_N]^+^** ***m*/*z* 262.22**
**B**	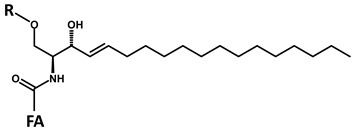	**d18:1-4** **C_18_H_37_NO_2_** **MW 299.50** **O”** **C_18_H_34_N]^+^** ***m*/*z* 264.22**
**C**	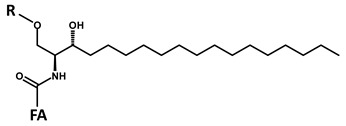	**d18:0** **C_18_H_39_NO_2_** **MW 301.52** **O”** **C_18_H_36_N]^+^** ***m*/*z* 266.22**
**D**	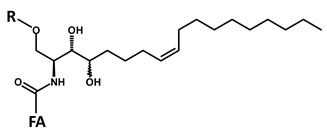	**t18:1-8** **C_18_H_37_NO_3_** **MW 315.50** **O”** **C_18_H_34_NO]^+^** ***m*/*z* 280.22**
**E**	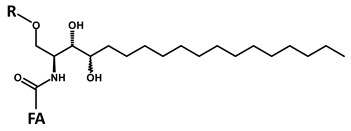	**t18:0** **C_18_H_39_NO_3_** **MW 317.52** **O”** **C_18_H_36_NO]^+^** ***m*/*z* 282.22**
	R = H (ceramide); R = Hex (cerebroside); FA = fatty acid residue Fragment O” = [LCB − 2 H_2_O] × H^+^	

**Table 2:** Levels of two specific ceramides in three almond and nine pistachio samples.

Sample ID	A1	A2
**M1**	13.65	n.d.
**M2**	22.48	n.d.
**M3**	36.47	n.d.
**P1**	39.97	13.64
**P2**	17.37	2.87
**P3**	14.69	4.53
**P4**	14.35	3.63
**P5**	15.95	4.04
**P6**	17.26	4.09
**P7**	19.40	3.74
**P8**	22.24	7.13
**P9**	12.52	2.85

A1: RT 8.5 min; MH^+^ 715; A2: RT 9.1 min; MH^+^ 699; Results in µg/g (expressed as Cer12:0-Glc equivalents); n.d.: not detected.

**Table 3:** Pistachio and almond samples examined in this study. The first column refers to the blinded analysis code and the second column to the sample code.

**ID**	**Sample**	**Nut Product**	**Origin**	**Characteristics**
Pistachio (*Pistacia vera* L.)				
**P1**	P7	Shelled	Bronte DOP (Sicily, Italy)	Not roasted, not salted
**P2**	P8	Shell	USA	Roasted, salted
**P3**	P9	Shell	Non EU	Organic, roasted, not salted
**P4**	P10	Shell	Iran	Not roasted, not salted
**P5**	P11	Shell	USA	Roasted, not salted
**P6**	P12	Shelled	Noberasco	Not roasted, not salted
**P7**	P13	Flour	Iran	
**P8**	P14	Flour	Italy	
**P9**	P15	Flour	Italy	
**ID**	**Sample**	**Nut Product**	**Origin**	**Characteristics**
Almond *(Prunus dulcis* Mill.)				
**M1**	A6	Shelled	California (USA)	Died
**M2**	A7	Shelled	California (USA)	Dried
**M3**	A8	Shelled	California (USA)	Dried

## Supplementary Materials

The following are available online at https://www.mdpi.com/2304-8158/9/2/110/s1. Figure S1: High-resolution mass spectrometry analysis for the confirmation of the unexpected ceramide species in the extract of a pistachio cultivar. Table S1: High-resolution measurement of fragment ion spectra of the four main ceramide species. Figure S2: High-resolution molecular (a, c, e) and fragment ion spectra (b, d, f) of triglycerides. Figure S3: Chromatographic traces of the Par263 and Par264 scans for a representative extract. Figure S4: Formation of protonated diglyceride and acylium fatty acid fragments from triglycerides. Figure S5: Plot of the relationship of chromatographic retention vs. molecular size for ceramides.

## Appendix A

Re-arranged form of the standard equation for energy transfer in Collision Induced Dissociation.

In Collision Induced Dissociation of ions, the relation of centre-of-mass, or “effective” (E _CM_) to laboratory-frame or “nominal” (E _lab_) collision energy depends:

- On the mass of the resting target gas in the collision cell (m _TAR_) and
- On the mass (*m*/*z*) of the impinging (singly-charged) precursor ion (m _PAR_),

according to the standard Equation (1).

E _CM_ = E _lab_ × m _TAR_/(m _TAR_ + m _PAR_)

For a fixed collision gas employed (e.g., m _TAR_, nitrogen, MW 28, or argon, MW 40), and for each specific value of “laboratory frame” collision energy, the “centre-of-mass” collision energy decreases as the *m*/*z* value of the precursor ions increase. The variation is not linear, either with the *m*/*z* of the precursor ion, or with the value of the “laboratory frame” collision energy.

Standard algebraic passages allow re-formulating Equation (1) to the linear working Equation (2):

E _lab_ = E _CM_ + E _CM_ × m _PAR_/m _TAR._

This linear equation allows to “back”-calculate the necessary laboratory frame collision voltage at each value of *m*/*z* of the precursor ion corresponding to a specific constant value of centre-of-mass collision energy.

## References

[1] Hawker J.S., Buttrose M.S. (1980). Development of the Almond Nut (*Prunus dulcis* (Mill.) DA Webb). Anatomy and Chemical Composition of Fruit Parts from Anthesis to Maturity. Ann. Bot..

[2] Summo C., Palasciano M., De Angelis D., Paradiso V.M., Caponio F., Pasqualone A. (2018). Evaluation of the chemical and nutritional characteristics of almonds (*Prunus dulcis* (Mill). DA Webb) as influenced by harvest time and cultivar. J. Sci. Food Agric..

[3] Alasalvar C., Bolling B.W. (2015). Review of nut phytochemicals, fat-soluble bioactives, antioxidant components and health effects. Br. J. Nutr..

[4] Wang D.D., Toledo E., Hruby A., Rosner B.A., Willett W.C., Sun Q., Razquin C., Zheng Y., Ruiz-Canela M., Guasch-Ferré M. (2017). Plasma ceramides, mediterranean diet, and incident cardiovascular disease in the PREDIMED trial (prevención con dieta mediterránea). Circulation.

[5] Neeland I.J., Singh S., McGuire D.K., Vega G.L., Roddy T., Reilly D.F., Castro-Perez J., Kozlitina J., Scherer P.E. (2018). Relation of plasma ceramides to visceral adiposity, insulin resistance and the development of type 2 diabetes mellitus: The Dallas Heart Study. Diabetologia.

[6] Fang F., Ho C.T., Sang S., Rosen R.T. (2005). Determination of sphingolipids in nuts and seeds by a single quadrupole liquid chromatography-mass spectrometry method. J. Food Lipids.

[7] Michaelson L.V., Napier J.A., Molino D., Faure J.D. (2016). Plant sphingolipids: Their importance in cellular organization and adaption. Biochim. Biophys. Acta.

[8] Miraliakbari H., Shahidi F. (2008). Antioxidant activity of minor components of tree nut oils. Food Chem..

[9] Paroni R., Dei Cas M., Rizzo J., Ghidoni R., Montagna M.T., Rubino F.M., Iriti M. (2019). Bioactive phytochemicals of tree nuts. Determination of the melatonin and sphingolipid content in almonds and pistachios. J. Food Compos. Anal..

[10] Sang S., Kikuzaki H., Lapsley K., Rosen R.T., Nakatani N., Ho C.T. (2002). Sphingolipid and other constituents from almond nuts (*Prunus amygdalus* Batsch). J. Agric. Food Chem..

[11] Rubino F.M., Zecca L., Sonnino S. (1994). Characterization of a Complex Mixture of Ceramides by Fast-Atom-Bombardment and Precursor and Fragment Analysis Tandem Mass-Spectrometry. Biol. Mass Spectrom..

[12] Reisberg M., Arnold N., Porzel A., Neubert R.H.H., Dräger B. (2017). Production of Rare Phyto-Ceramides from Abundant Food Plant Residues. J. Agric. Food Chem..

[13] Motta S., Sesana S., Ghidoni R., Monti M. (1995). Content of the different lipid classes in psoriatic scale. Arch. Dermatol. Res..

[14] Gaudin K., Chaminade P., Baillet A. (2002). Structure-retention diagrams of ceramides established for their identification. J. Chromatogr. A.

[15] Murphy R.C., James P.F., McAnoy A.M., Krank J., Duchoslav E., Barkley R.M. (2007). Detection of the abundance of diacylglycerol and triacylglycerol molecular species in cells using neutral loss mass spectrometry. Anal. Biochem..

[16] Goodacre R., Vaidyanathan S., Bianchi G., Kell D.B. (2002). Metabolic profiling using direct infusion electrospray ionisation mass spectrometry for the characterisation of olive oils. Analyst.

[17] Gorrepati K., Balasubramanian S., Chandra P. (2015). Plant based butters. J. Food Sci. Technol..

[18] Yildirim A.N., Akinci-Yildirim F., Şan B., Sesli Y. (2016). Total Oil Content and Fatty Acid Profile of some Almond (Amygdalus communis L.) Cultivars. Pol. J. Food Nutr. Sci..

[19] Moayedi A., Rezaei K., Moini S., Keshavarz B. (2011). Chemical compositions of oils from several wild almond species. J. Am. Oil Chem. Soc..

[20] Fernandes G.D., Gómez-Coca R.B., Pérez-Camino M.D.C., Moreda W., Barrera-Arellano D. (2017). Chemical Characterization of Major and Minor Compounds of Nut Oils: Almond, Hazelnut, and Pecan Nut. J. Chem..

[21] Evoli L.D., Lucarini M., Gabrielli P., Aguzzi A., Lombardi-boccia G. (2015). Nutritional Value of Italian Pistachios from Bronte (Pistacia vera, L.), Their Nutrients, Bioactive Compounds and Antioxidant Activity. Food Nutr. Sci..

[22] Alasalvar C., Shahidi F. (2008). Tree Nuts: Composition, Phytochemicals, and Health Effects: An Overview. Tree Nuts: Composition, Phytochemicals, and Health Effects.

[23] Valsecchi M., Mauri L., Casellato R., Ciampa M.G., Rizza L., Bonina A., Bonina F., Sonnino S. (2012). Ceramides as possible nutraceutical compounds: Characterization of the ceramides of the moro blood orange (Citrus sinensis). J. Agric. Food Chem..

[24] Liang L., Wu X., Zhu M., Zhao W., Li F., Zou Y., Yang L. (2012). Chemical composition, nutritional value, and antioxidant activities of eight mulberry cultivars from China. Pharmacogn. Mag..

[25] Esteki M., Ahmadi P., Vander Heyden Y., Simal-Gandara J. (2019). Fatty acids-based quality index to differentiate worldwide commercial pistachio cultivars. Molecules.

[26] Bernardi N., Benetti G., Campolongo G., Ferrari G., Palermo R., Vranic B. (2014). Preliminary Assessment of Vegetable Oil Adulteration of Pistachio Paste by near Infrared Spectroscopy. NIR News.

[27] Eksi-Kocak H., Mentes-Yilmaz O., Boyaci I.H. (2016). Detection of green pea adulteration in pistachio nut granules by using Raman hyperspectral imaging. Eur. Food Res. Technol..

[28] Sezer B., Apaydin H., Bilge G., Boyaci I.H. (2018). Detection of Pistacia vera adulteration by using laser induced breakdown spectroscopy. J. Sci. Food Agric..

[29] Çavuş F., Us M.F., Güzelsoy N.A. Assesing Pistachio Nut (Pistacia vera L.) Adulteration with Green Pea (Pisum sativum L.) by Untargeted Liquid Chromatography-(Quadrupole-Time of Flight)-Mass Spectrometry Method and Chemometrics. https://dergipark.org.tr/en/pub/bursagida/issue/40169/477784.

[30] Canela N., Herrero P., Mariné S., Nadal P., Ras M.R., Rodríguez M.Á., Arola L. (2016). Analytical methods in sphingolipidomics: Quantitative and profiling approaches in food analysis. J. Chromatogr. A.

[31] Markham J.E., Jaworski J.G. (2007). Rapid measurement of sphingolipids from Arabidopsis thaliana by reversed-phase high-performance liquid chromatography coupled to electrospray ionization tandem mass spectrometry. Rapid Commun. Mass Spectrom..

[32] Dalmau N., Jaumot J., Tauler R., Bedia C. (2015). Epithelial-to-mesenchymal transition involves triacylglycerol accumulation in DU145 prostate cancer cells. Mol. Biosyst..

