# Supplementary material for: Discovery of Unexpected Sphingolipids in Almonds and Pistachios with an Innovative Use of Triple Quadrupole Tandem Mass Spectrometry
Source: Foods. 2020 Jan 21;9(2):110. doi: 10.3390/foods9020110 (PMC7074565; doi:10.3390/foods9020110)
Supplement: Supplementary file 1 [file foods-09-00110-s001.pdf]

Discovery of unexpected sphingolipids in almonds and pistachios with an innovative use of triple quadrupole tandem mass spectrometry

Supplementary information

Figure S1. High-resolution mass spectrometry analysis for the confirmation of the unexpected ceramide species in the extract of a pistachio cultivar. A1 (left) Molecular ion region of protonated A1 and (right) fragment ions spectrum (CE 30 eV). A2 (left) Molecular ion region of protonated A2 and (right) fragment ions spectrum (CE 30 eV).

Table S1 High-resolution measurement of fragment ion spectra of the four main ceramide species in two almond and two pistachio extracts[a].

Figure S2. High-resolution molecular (a, c, e) and fragment ion spectra (b, d, f) of triglycerides as ammonium adducts. (a, b) TG 54:5 (m/z 898.7771). (c,d) TG 54:4 (m/z 900.7925). (e,f) TG 54:3 (m/z 902.8055).

Figure S3. Chromatographic traces of the Par263 and Par264 scans for a representative pistachio extract.

Figure S4. Formation of protonated diglyceride and acylium fatty acid fragments from ammoniated triglycerides by Collision Activated Decomposition.

Figure S5. Plot of the relationship of chromatographic retention vs. molecular size for the series of ceramides with d18:1D4 sphingosine long-chain base (LCB) and saturated straight-chain even-Carbon fatty acids (FA).

Figure S1. High-resolution mass spectrometry analysis for the confirmation of the unexpected ceramide species in the extract of a pistachio cultivar. A1 (left) Molecular ion region of protonated A1 and (right) fragment ions spectrum (CE 30 eV). A2 (left) Molecular ion region of protonated A2 and (right) fragment ions spectrum (CE 30 eV).

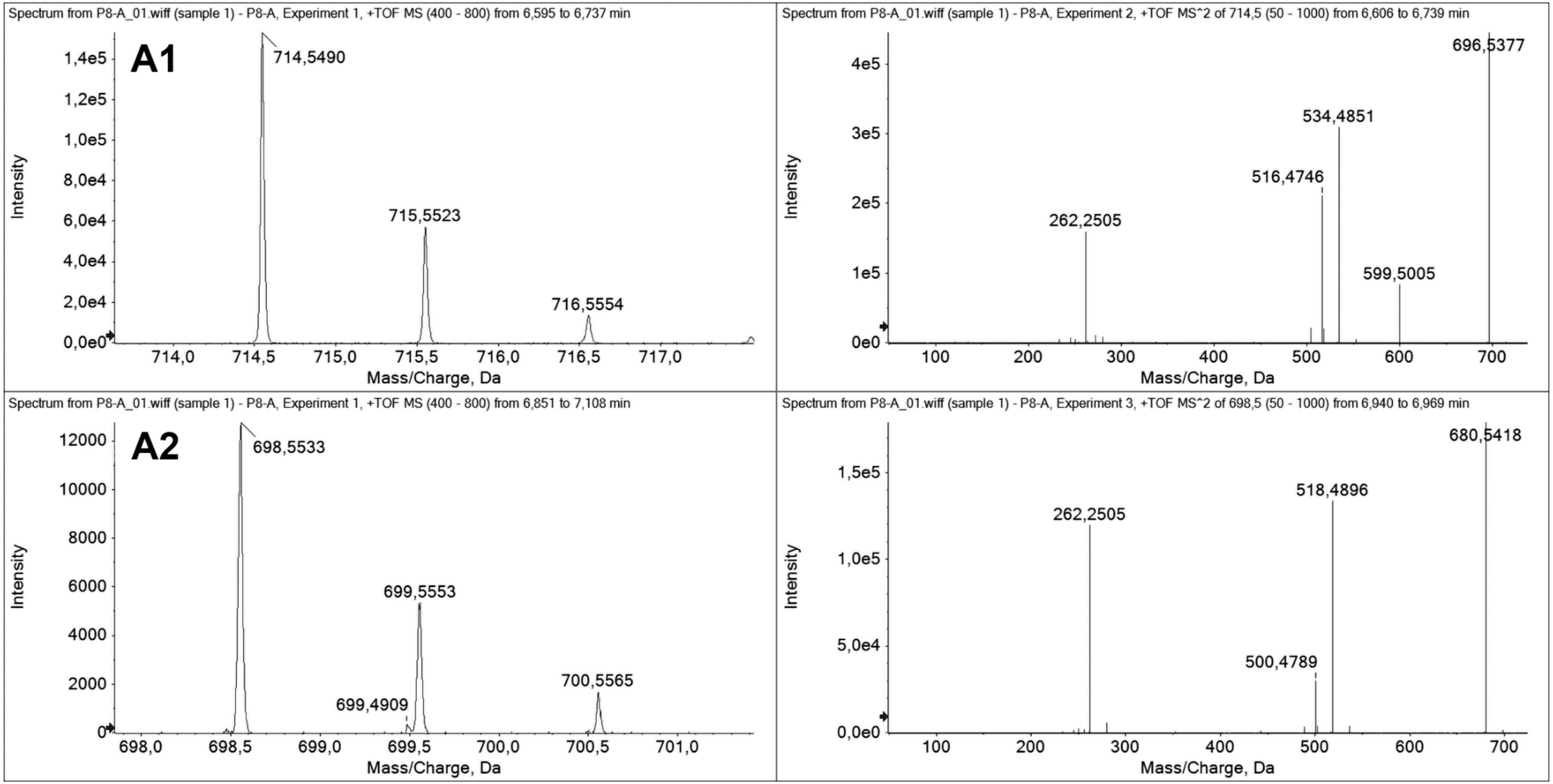

TOF-MS analysis (250 ms accumulation time, DP 50 eV) and TOF-MS/MS analysis (100 ms accumulation time, DP 50 eV, CE 30 eV).

Table S1. High-resolution measurement of fragment ion spectra of the four main ceramide species in two almond and two pistachio extracts.

cpd	ion_id	calc for	mz	M1 mz	M2 mz	P8 mz	P9 mz	mean mz	M1 ppm	M2 ppm	P8 ppm	P9 ppm
A1 [a]	MH+	C40 H76 N1 O9	714.5520	714.5436	714.5464	714.5490	714.5450	714.5460	-11.8	-7.8	-4.2	-9.8
A1	-H2O	C40 H74 N1 O8	696.5414	696.5329	696.5359	696.5376	696.5347	696.5353	-12.3	-8.0	-5.5	-9.7
A1	-C6H12O6	C34 H64 N1 O3	534.4886	534.4824	534.4846	534.4821	534.4833	534.4831	-11.6	-7.5	-12.2	-10.0
A1	-H2O-C6H12O6	C34 H62 N1 O2	516.4781		516.4717	516.4746	516.4722	516.4728		-12.3	-6.7	-11.3
A1	-C6H12O6-H2CO	C33 H62 N1 O2	504.4781	504.4718	504.4771	504.4748	504.4726	504.4741	-12.4	-1.9	-6.5	-10.8
A1	O″ B18d:2 - 2 H2O	C18 H32 N1 O0	262.2535	262.2505	262.2519	262.2505	262.2504	262.2508	-11.3	-6.0	-11.3	-11.7
A2 [a]	MH+	C40 H76 N1 O8	698.5571	698.5485	698.5475	698.5535	698.5516	698.5503	-12.3	-13.7	-5.1	-7.9
A2	-H2O	C40 H74 N1 O7	680.5465	680.5381	680.5391	680.5420	680.5412	680.5401	-12.4	-10.9	-6.7	-7.8
A2	-C6H12O6	C34 H64 N1 O2	518.4937	518.4864	518.4881	518.4895	518.4889	518.4882	-14.1	-10.8	-8.1	-9.3
A2	-H2O-C6H12O6	C34 H62 N1 O1	500.4831	500.4767	500.4769	500.4788	500.4784	500.4777	-12.9	-12.5	-8.7	-9.5
A2	O′ B18d:2 - H2O	C18 H34 N1 O1	280.2640		280.2605			280.2605		-12.6		
A2	O″ B18d:2 - 2 H2O	C18 H32 N1 O0	262.2535	262.2497	262.2510	262.2504	262.2508	262.2505	-14.4	-9.4	-11.7	-10.2
Cer12:0 [a]	MH+	C30 H60 N1 O3	482.4573	482.4513	482.4521	482.4540	482.4514	482.4522	-12.5	-10.8	-6.9	-12.3
Cer12:0	- H2O	C30 H58 N1 O2	464.4468	464.4411	464.4423	464.4426	464.4415	464.4419	-12.2	-9.6	-8.9	-11.3
Cer12:0	O′ B18d:1 - H2O	C18 H36 N1 O1	282.2797	282.2760	282.2772	282.2766	282.2757	282.2764	-13.1	-8.8	-10.9	-14.1
Cer12:0	O″ B18d:1 - 2 H2O	C18 H34 N1 O0	264.2691	264.2662	264.2671	264.2659	264.2656	264.2662	-11.1	-7.7	-12.2	-13.3
Cer12:0	O‴ B18d:1 - H2O -H2CO	C17 H34 N1 O0	252.2691	252.2658	252.2669	252.2665	252.2653	252.2661	-13.2	-8.8	-10.4	-15.2
HexCer12:0 [a]	MH+	C36 H70 N1 O8	644.5101	644.4935	644.5023	644.5071	644.5046	644.5019	-25.8	-12.2	-4.7	-8.6
HexCer12:0	MH+ -H2O	C36 H68 N1 O7	626.4996	626.4924	626.4945	626.4957	626.4927	626.4938	-11.5	-8.1	-6.2	-11.0
HexCer12:0	MH+ Cer12:0	C30 H60 N1 O3	482.4573	482.4502				482.4502	-14.8			
HexCer12:0	-C6H10O5	C30 H58 N1 O2	464.4468	464.4416	464.4431	464.4431	464.4414	464.4423	-11.1	-7.9	-7.9	-11.5
HexCer12:0	-C6H10O5 - H2O	C30 H56 N1 O1	446.4362	446.4313	446.4323	446.4325	446.4313	446.4319	-11.0	-8.7	-8.3	-11.0
HexCer12:0	MH+ -Glc - H2CO	C29 H56 N1 O1	434.4362	434.4306				434.4306	-12.9			
HexCer12:0	O′ B18d:1 - H2O	C18 H36 N1 O1	282.2797	282.2756		282.2768	282.2753	282.2759	-14.5		-10.2	-15.6
HexCer12:0	O″ B18d:1 - 2 H2O	C18 H34 N1 O0	264.2691	264.2661	264.2671	264.2661	264.2656	264.2662	-11.4	-7.7	-11.4	-13.3
HexCer12:0	O‴ B18d:1 - H2O -H2CO	C17 H34 N1 O0	252.2691	252.2649			252.2649	252.2649	-16.7			-16.7

[a]: A1 and A2 are the natural components of the extracts; Cer12:0 and HexCer12:0 (Hex = Glucose) are the two added internal standards.

Figure S2. High-resolution molecular (a, c, e) and fragment ion spectra (b, d, f) corresponding to triglycerides as ammonium adducts. (a, b) TG 54:5 (m/z 898.7771). (c,d) TG 54:4 (m/z 900.7925). (e,f) TG 54:3 (m/z 902.8055).

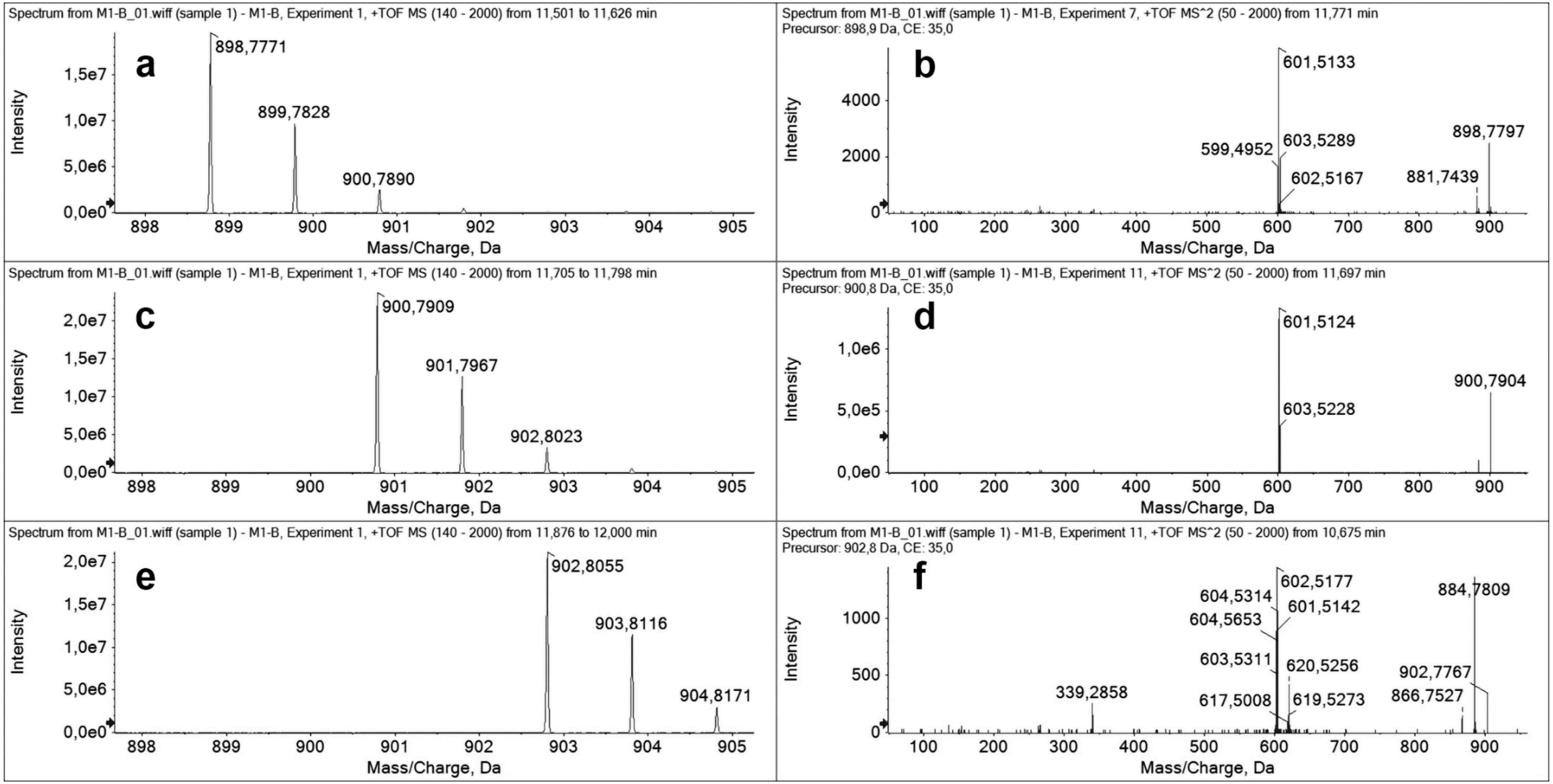

TOF-MS analysis (250 ms accumulation time, DP 50 eV) and TOF-MS/MS analysis (100 ms accumulation time, DP 50 eV, CE 35 eV).

In the fragment ion spectrum of m/z 900, observed are the (protonated dehydrated) diglyceride fragments (*m/z 601* and 603, 3:1 ratio) due to the alternative competitive loss of oleic and linoleic acid as ammoniated neutral species.

Figure S3. Chromatographic traces of the Par263 and Par264 scans for a representative pistachio extract.

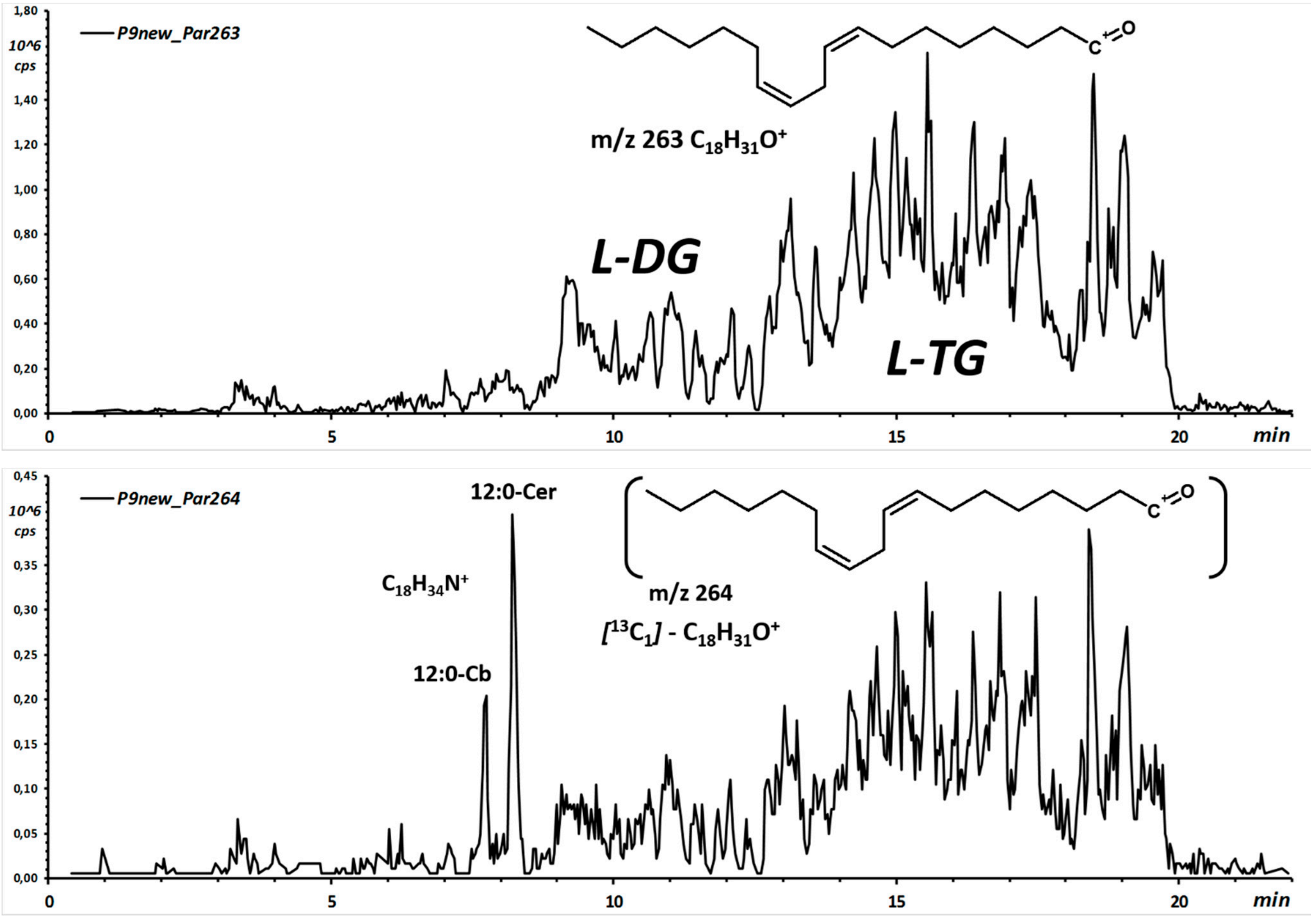

From 13 to 20 min the Par263 scan detects triglycerides that contain *all*-^{12}C linoleic acid (L-TG) by formal loss of the corresponding ammoniated fatty acid. In the same time frame, the Par264 scan detects triglycerides that contain $^{13}C_1$ linoleic acid isotopomers. As apparent from the values of the vertical scale, the signals are in an *approx.* 4:1 intensity ratio that is expected for the occurrence of the ^{13}C isotopomer in the isotope cluster of ammoniated triglyceride precursors that detach an ammoniated fatty acid.

Figure S4. General scheme of the formation of protonated diglyceride and acylium fatty acid fragments from ammoniated triglycerides by Collision Activated Decomposition.

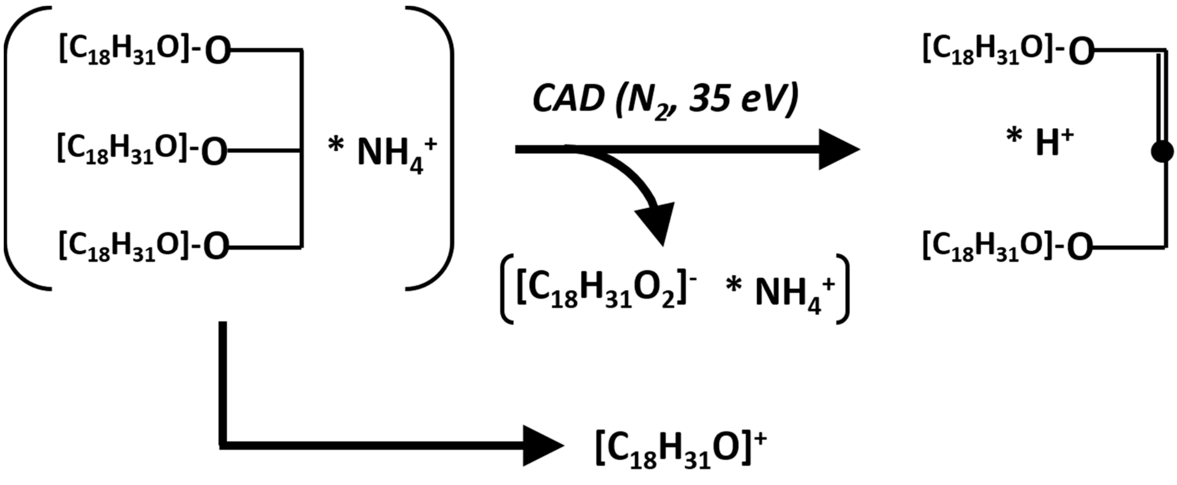

Figure S5. Plot of the relationship of chromatographic retention vs. molecular size for the series of ceramides with d18:1D4 sphingosine long-chain base (LCB) and saturated straight-chain even-Carbon fatty acids (FA).

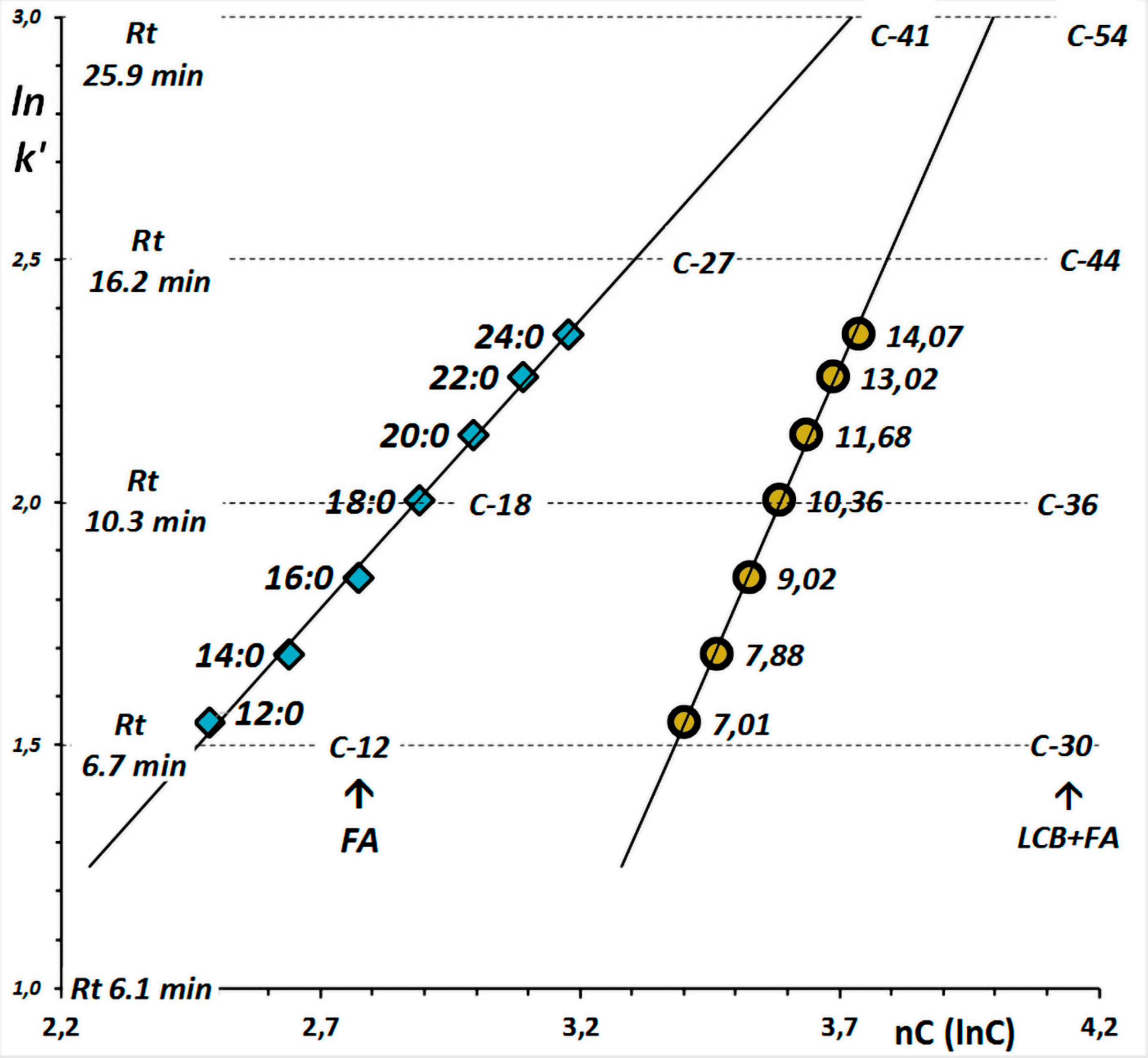

Retention (y axis) is calculated as the capacity factor (*k'*) and reported as its natural logarithm, ln(*k'*). Molecular size (x axis) is reported as the natural logarithm of both the sole number of Carbons in the FA (blue ◇), and the total number of Carbon in the ceramide (18 carbons of the LCB + n Carbons of the FA; yellow ⭘). On y axis are also reported for some ln(*k'*) the corresponding retention time (min). Linear regression equations are y=1,1921x-1,4391 R^2=0,9969 and y=2,4496x-6,787 R^2=0,9974 for the number of fatty acid Carbon and for the total number of Carbons, respectively.
